# Real‐Life Application of a Point‐of‐Care Biosensor for Phenylalanine in Patients With Phenylketonuria

**DOI:** 10.1002/jimd.70187

**Published:** 2026-04-16

**Authors:** Corentin Gondrand, Anna T. Reischl‐Hajiabadi, Estelle Bonedeau, Nicolas Weiß, Gerd Schneider, Maurice Fahn, Frederic Myers, Philipp Minel Städle, Kathrin Jeltsch, Georg F. Hoffmann, Jürgen G. Okun, Dorothea Haas, Sven F. Garbade, Kai Johnsson, Thomas Opladen

**Affiliations:** ^1^ Department of Chemical Biology Max Planck Institute for Medical Research Heidelberg Germany; ^2^ Medical Faculty of Heidelberg, Department of Pediatrics, Division of Pediatric Neurology and Metabolic Medicine Heidelberg University Heidelberg Germany; ^3^ Phellow Seven GmbH Heidelberg Germany; ^4^ Frederic Myers Consulting (JERY) Reutlingen Germany; ^5^ École Polytechnique Fédérale de Lausanne Institute of Chemical Sciences and Engineering, NCCR in Chemical Biology Lausanne Switzerland

**Keywords:** at‐home test, biosensor, phenylalanine hydroxylase deficiency, Phenylketonuria, PKU, point‐of‐care testing

## Abstract

Phenylketonuria (PKU) is a rare metabolic disorder causing elevated phenylalanine (PHE) levels requiring lifelong dietary or pharmacological management and regular monitoring. Current PHE monitoring methods, such as tandem mass spectrometry (FIA‐MS/MS), are laboratory and sample transport‐dependent, leading to delays in obtaining results. This highlights the need for accessible point‐of‐care (PoC) solutions. Building on a previously developed bioluminescence‐based PHE quantification method, we created a PoC PHE sensor integrated with a smartphone app (*phenyx* app) for real‐time monitoring and data sharing. In a clinical study with PKU patients, we evaluated the reliability of the sensor and the app by comparing at‐home PHE measurements with dried blood samples (DBS) analyzed using the gold standard FIA‐MS/MS. We also assessed time differences between sampling and results, sensor practicality, and user experience. Forty‐seven PKU patients (median age 10.0 years, range 0.7–53.4) performed one supervised and four at‐home measurements using the PHE sensor, alongside DBS sampling for FIA‐MS/MS. The sensor showed higher PHE values than FIA‐MS/MS, with a mean difference of 84.8 μmol/L (*p* = 0.0017). Variability was influenced by test supervision (clinic vs. home) and test kit age (greater difference in older batches, *p* = 0.001). Usability of the PHE sensor and *phenyx* app was rated favorably; all patients preferred the PHE sensor over laboratory testing. PoC measurement using the PHE sensor and *phenyx* app is feasible, reliable, and provides rapid results comparable to FIA‐MS/MS, representing a key step toward patient self‐testing.

**Trial Registration:** NCT05998109, NCT06940193, DRKS00031972.

**Abbreviations**
BH_4_
: tetrahydrobiopterin
BRET: bioluminescent resonance energy transfer
DBS: dried blood spot
FIA‐MS/MS: Flow injection analysis–tandem mass spectrometry
FRET: fluorescence resonance energy transfer
LME: linear mixed‐effects model
NADPH: nicotinamide adenine dinucleotide phosphate
PAH: phenylalanine hydroxylase
PDH: phenylalanine dehydrogenase
PHE: phenylalanine
PKU: phenylketonuria
PoC: point‐of‐care

## 1 Introduction

Phenylketonuria (PKU; OMIM #261600) is one of the most common inherited metabolic diseases with a birth prevalence of approximately 1 in 10 000. It is an autosomal recessive inherited disorder affecting the conversion of the essential amino acid phenylalanine (PHE) to tyrosine due to impaired activity of phenylalanine hydroxylase (PAH; EC 1.14.16.1). Under physiological conditions, PAH converts phenylalanine into tyrosine using the cofactor tetrahydrobiopterin (BH_4_). In PKU, the deficient *PAH* gene leads to accumulation of PHE in blood and all body fluids. PHE is thus a reliable biomarker for diagnosis of PKU, for example, in newborn screening, but also for lifelong disease monitoring [1, 2, 3, 4].

If untreated, PKU causes severe intellectual impairment and additional neurological symptoms. Therefore, affected patients require lifelong dietary or drug treatment to maintain PHE concentrations within an age‐appropriate target range [2, 3, 4, 5, 6]. PHE levels can be measured in dried blood spots (DBS) or plasma by tandem mass spectrometry (FIA‐MS/MS), high‐performance liquid chromatography, or amino acid analyzer [4, 5, 6, 7]. While these methods are effective, they are time‐consuming, expensive, and limited to specialized laboratories. When collecting DBS and sending DBS cards to the laboratory, the delivery and laboratory turnaround times cause a delay of several days in receiving the test results, highlighting the need for adaptable test formats. Point‐of‐care (PoC) and at‐home tests provide fast results outside traditional laboratory settings. These tests enhance accessibility, affordability, and patient compliance, especially for those requiring frequent monitoring, such as PKU patients.

Although no commercial solution is yet available, numerous studies have developed quantitative methods for measuring PHE concentrations. These include electrochemical biosensors based on voltammetry, amperometry, potentiometry, or impedimetric, which can be integrated into handheld devices [8, 9, 10, 11]. Optical biosensors including colorimetric approaches such as gold nanoparticles‐integrated paper tests [12] or enzymatic tests using phenylalanine ammonia‐lyase (PheCheck) [13], as well as fluorimetric methods such as fibre‐optic sensors [14], and luminometric approaches such as chemiluminescent microfluidic assays [15] or UV‐activated smartphone‐adapted fluorescent sensors [16]. Additional solutions include mass‐sensitive immunosensors [17] and surface plasmon resonance chips [18].

While these technologies show significant promises over the standard of care in terms of simplicity, speed, and cost, the transition from laboratory prototypes to practical, patient‐friendly solutions is challenging. Many methods are validated only in simplified matrices such as blood serum [8, 9, 12, 18], require specialized equipment [14, 15, 18], or multiple‐step workflows [16], restricting their applicability in PoC settings. Beyond the PheCheck method [13], which is currently under evaluation in a clinical trial (NCT05998109) and the recently published at‐home PHE measurement system [19] (NCT06940193), the performance of other technologies in real‐world conditions ‐ particular when used by patients or caregivers in home settings, remains largely unassessed.

We had previously developed a bioluminescence‐based method enabling PHE quantification at PoC from whole blood [20]. Results from patient samples agreed with those obtained with the standard of care, FIA‐MS/MS. Based on this method, we designed a test kit, referred to as PHE sensor, designed for at‐home self‐testing by PKU patients. We integrated this PHE sensor with a smartphone readout and smartphone health application, the *phenyx* app, to enable real‐time PHE level monitoring and data sharing with healthcare providers. This integration aims to support remote patient monitoring and personalized care, identify trends in PHE levels, flag issues like poor dietary compliance, medication errors, or fluctuations during illness, and enable recommendations for adjustments to treatment or diet.

The primary objective was to evaluate the reliability of the PHE sensor and *phenyx* app when used by PKU patients, in a real‐world, at‐home environment. Secondary objectives included the comparison of sampling‐to‐result times with FIA‐MS/MS and the assessment of practicability of the PHE sensor and *phenyx* app.

## 2 Methods

### 2.1 Study Design

This single‐center study (German Clinical Trials Register; trial registration ID: DRKS00031972) was conducted at Heidelberg University Hospital from February 2024 to April 2025 and approved by the local ethics committee (University Hospital Heidelberg, Germany; application number: S‐109/2023). The study was open to all interested patients with genetically confirmed PKU. The study followed routine PHE monitoring as recommended by the German consensus guidelines for PKU (updated version 2025 currently under review, AWMF 027 ‐ 002). Written informed consent was obtained from all participants or their legal guardians. None of the patients were treated with Pegvaliase, and one pregnant woman received sapropterin.

PHE concentrations were measured in capillary blood at PoC using the PHE sensor, with parallel measurements in DBS obtained via capillary puncture and analyzed by FIA‐MS/MS at five predefined time points. The parallel measurements required only 5 μL of additional blood without extra pricks. The study adhered to the routine monitoring schedule, which recommends biweekly PHE monitoring for patients aged 0–12 years, and monthly monitoring for patients aged 13 years and older. This resulted in a study duration of approximately 2 and 4 months, respectively. Following an initial onboarding appointment at the outpatient clinic, including training and first supervised parallel measurements, the remaining four measurements were conducted independently at home (Table S1). The study was conducted sequentially in five groups due to the production of small test batches and the available smartphones. The age and sex distribution of the five groups is presented in Table 1. The date of receipt of DBS in the laboratory and the date of reporting the results were obtained from the laboratory information system.

**TABLE 1:** Age and sex distribution of the study groups.

	Total	Group 1	Group 2	Group 3	Group 4	Group 5
*N*	47	10	14	10	7	6
Male/female	17/30	4/6	8/6	2/8	0/7	3/3
Median age in years (min‐max)	10.0 (0.7–53.4)	11.9 (1.8–38.9)	5.1 (1.7–11.8)	21.9 (9.7–44.3)	44.4 (28.8–53.4)	5.8 (0.7–11.9)

### 2.2 Questionnaire

At the end of the study, participants or caregivers completed a 23‐item questionnaire regarding their user experience of the PHE sensor and the *phenyx* app. The survey covered usability, handling issues, confidence in handling, perceived effort compared to laboratory testing, preference for PHE sensor use, and desired app features (Table S2).

### 2.3 PHE Sensor

**FIGURE 1:**
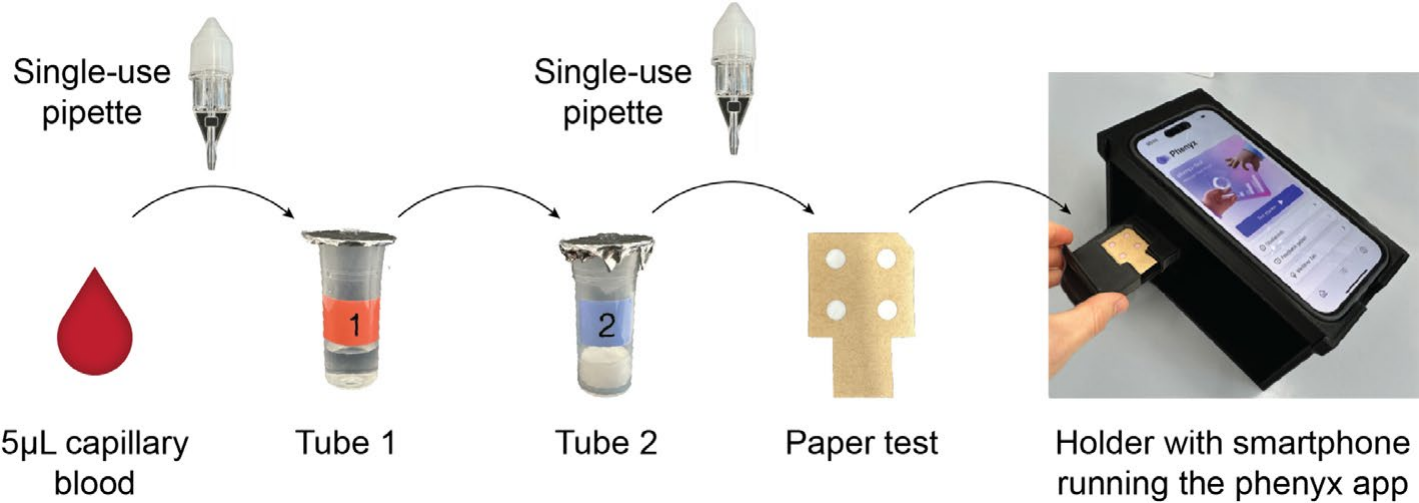
PHE sensor measurement process. A volume of 5 μL of blood obtained from a finger prick is collected with a single‐use pipette. The blood‐filled pipette is clicked onto tube 1. The content of tube 1 is then transferred into tube 2, followed by a 5‐min incubation. A drop from tube 2 is then applied on each detection zone of the paper test, resulting in the light emission of the NADPH sensor. The paper test is inserted into the drawer of the smartphone holder, where a smartphone running the *phenyx* app takes and analyses pictures of the test signal, calculates PHE concentration, and transmits the result to the medical team.

The PHE sensor was adapted from a bioluminescence‐based PHE PoC assay [20] developed at the Max Planck Institute for Medical Research. In this method, PHE is converted by an NADP^+^‐dependent phenylalanine dehydrogenase into NADPH, which is quantified by a bioluminescent NADPH sensor (Figure S1). The PHE sensor is a test kit containing a lancet, two single‐use pipettes, a tube containing buffer (tube 1), a tube with lyophilized reagents (tube 2), a paper test with four detection zones, and a smartphone holder (Figure 1). The *phenyx* app guided users through the test steps (summary of instructions in the Supporting Information).

Each smartphone used in the study was evaluated with the PHE sensor and results were compared to a reference iPhone 14 Pro to confirm color detection accuracy (Figure S2).

Reagents (tube 2 and test paper) were produced in batches of 100–150 units; each batch was calibrated using spiked and PKU patient whole blood samples, and the shelf‐life was shown to be at least 6 weeks at 4°C (Figure S3). For patients on biweekly testing schedules, all kits were supplied at once. Patients tested monthly received two kits during clinic visits, with subsequent tests shipped separately, which introduced logistical constraints. In addition, replacement kits occasionally had to be shipped at short notice (e.g., after handling issues), requiring the redistribution of older batches.

### 2.4 Digital Health Application *Phenyx* App

The study partner phellow seven GmbH developed the *phenyx* app (phenyx.de) for Apple iOS. In addition to the app for patients, a browser‐based application was developed for the supporting team of medical professionals, enabling real‐time assessment of patient input and lab results. All participants received an iPhone 14 Pro with the *phenyx* app pre‐installed, pre‐configured, and a unique onboarding QR code. The app guided patients step by step through the PHE sensor measurement process using videos.

For the PHE level quantification, images were taken with the iPhone 14 Pro main camera (exposure 1 s, white balance 8000 K, focus distance 0.0, ISO 5000). Nine images were taken at five‐second intervals, beginning 3:45 min after applying the sample to the paper test. Images were transferred through a secure channel to the server, where they were processed and averaged to calculate PHE concentration. If any error occurred during processing (e.g., quality check fails) the system would display an error message to the patient.

The quantification algorithm, adapted from the literature, recognizes the test spots, determines mean pixel intensities in blue and red channels, and calculates their ratio [21]. PHE level is calculated from this ratio in combination with the calibration data stored in the QR code of each test.

**FIGURE 2:**
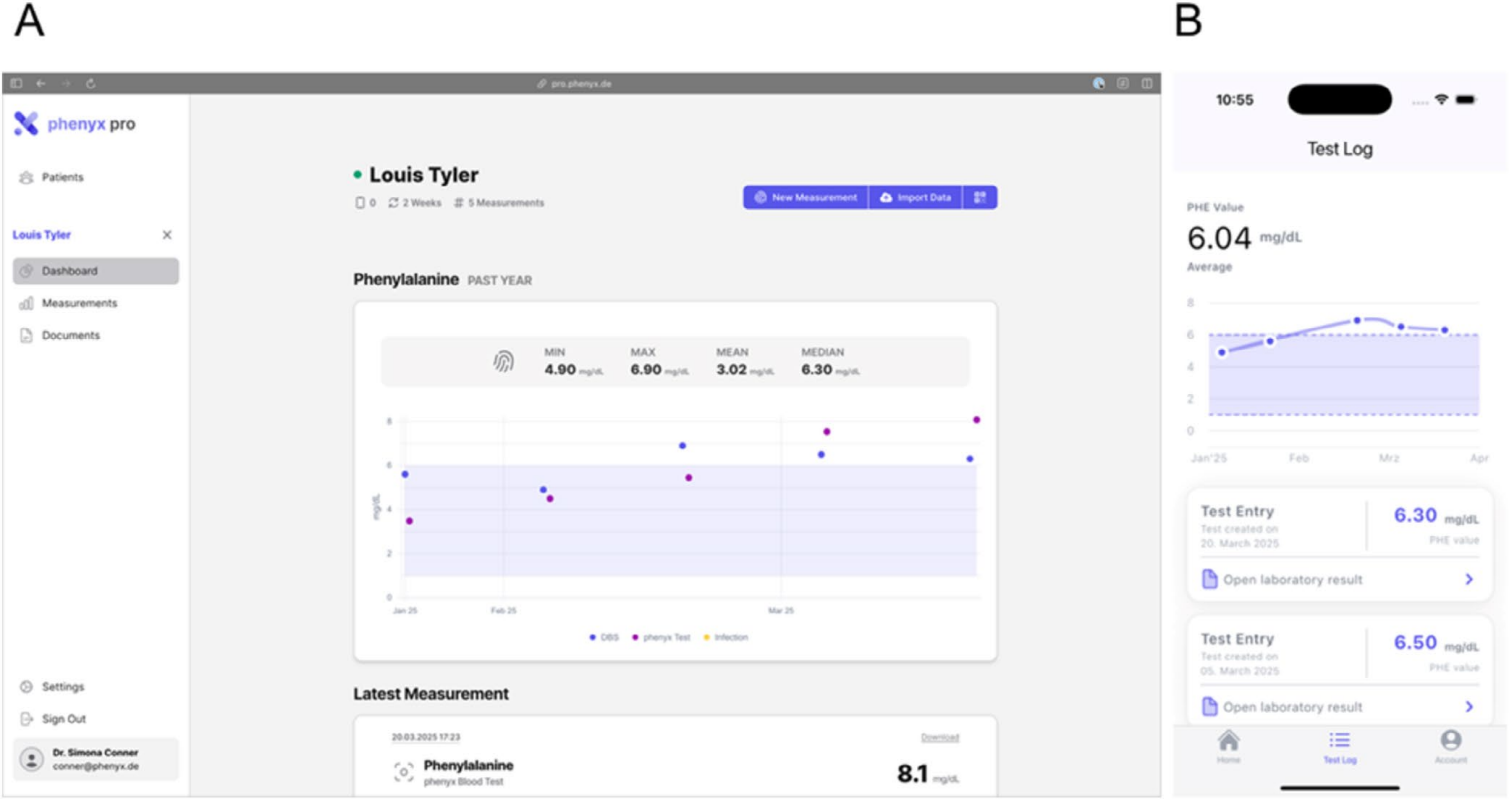
*phenyx* app dashboard. (A) Display of the *phenyx* app dashboard from the healthcare provider's perspective, presenting the PHE values from both the PHE sensor and DBS measurements, along with information on infection status and the patient's age‐specific reference range. (B) Display of the *phenyx* app dashboard from the patient's perspective, showing individual measurements, target range, as well as trends and averages. In the study, only the results from the DBS measurements were shown to the patients. The y‐axis is labeled from 0 to 8 mg/dL PHE (0 to 484 μmol/L), as the study was conducted using the unit mg/dL, which is common in German‐speaking countries. Please note that this information refers to a fictional patient and doctor and does not contain identifiable data from the study.

During the study, the PHE sensor results were not shared directly with patients to prevent self‐directed dietary changes prior to formal evaluation. By contrast, DBS results from FIA‐MS/MS were manually uploaded by the study team into the app and displayed as trends together with age‐specific target ranges for German‐speaking countries (< 6 mg/dL (< 360 μmol/L) for < 12 years and pregnant women; < 10 mg/dL (600 μmol/L) for < 15 years, < 15 mg/dL (900 μmol/L) for < 18 years, < 20 mg/dL (1200 μmol/L) for > 18 years) (Figure 2).

### 2.5 DBS PHE Quantification With FIA‐MS/MS

DBS samples were measured in the laboratory using the gold standard, FIA‐MS/MS [4, 5, 6, 7]. Because PHE concentrations in erythrocytes are 19% lower than in plasma, values were corrected using the slope of the 75th percentile from the quantile regression as previously described [7].

### 2.6 Statistical Analysis

Data were analyzed using descriptive and inferential statistical methods. All statistical analyses were conducted using R Version 4.5.0 and GraphPad Prism Version 10 and *p*‐values < 0.05 were considered statistically significant.

Exclusion of data from analyses was done if only one measurement (PHE sensor or DBS) was available for a given time point, kits had not been stored properly, plasma rather than DBS was used, testing was delayed for more than 2 weeks, or PHE sensor values were < 60 or > 2000 μmol/L (outside the quantifiable range of the PHE sensor).

The difference between the PHE sensor and FIA‐MS/MS was analyzed using a linear mixed‐effects (LME) model with a random intercept for the participants' IDs. The following predictor variables were analyzed in LME: group (study group 1–5), device (19 different devices), age (numeric), sex (female/male), test batch (six different batches), test batch age (numeric), test setting (supervised (1st test) versus at‐home tests (2nd–5th test)) and infection status (yes/no). Because of missing questionnaire data, the effect of the ranking of confidence during measurement [1, 2, 3, 4, 5, 6] answered in the questionnaire was evaluated with all predictor variables listed above, but with a lower sample size (*N* = 46, instead of *N* = 47). We used function lme() from R package “nlme” version 3.1‐168 to compute LME models. For categorical predictors, we report *p*‐values from analysis of variance tables with marginal sum of squares (function anova() from R package “nlme”), otherwise the regression coefficient *β* and the respective *p*‐value. To compute bias and tolerance limits of measurement differences between PHE sensor and FIA‐MS/MS, R package “SimplyAgree,” version 0.2.1 was applied.

## 3 Results

### 3.1 Study Population

Between February 2024 and April 2025, 47 patients (*N* = 17 male, *N* = 30 female) with genetically confirmed PKU and a median age of 10.0 years (range 0.7–53.4 years) participated across five groups (Table 1). Five test batches were produced with three batches used for group 1, two batches each for groups 2–4, and one batch for group 5. During the study, a total of 19 different iPhones were used.

A total of 194 parallel measurements using the PHE sensor and DBS (FIA‐MS/MS) were included in the analysis: study group 1: *N* = 45; group 2: *N* = 55; group 3: *N* = 44; groups 4 and 5: *N* = 25 each (Figure S4). When sub‐grouped according to age‐dependent target ranges, *N* = 116 were in the < 360 μmol/L range, *N* = 10 between 360 and 600 μmol/L, *N* = 14 between 600 and 900 μmol/L, and *N* = 54 between 900 and 1200 μmol/L. *N* = 50 measurement pairs were excluded due to out‐of‐range PHE sensor values (> 2000 μmol/L), the use of plasma instead of DBS, PHE sensor mishandling, improper kit storage and DBS transport issues (Table S3).

### 3.2 Comparison of PHE Levels From the PHE Sensor and DBS (FIA‐MS/MS)

**FIGURE 3:**
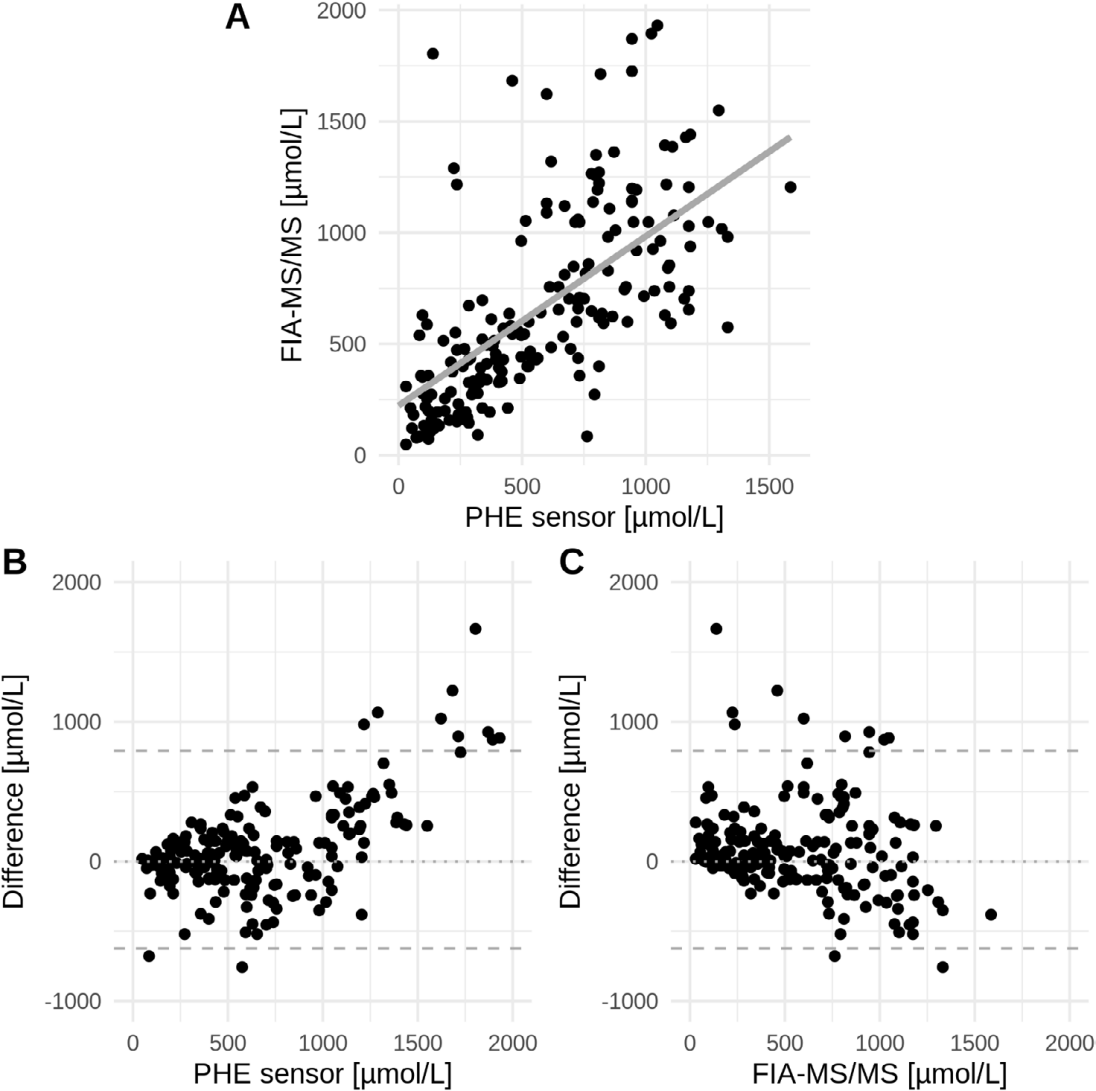
A: Scatterplot with of PHE sensor and DBS FIA‐MS/MS measurements. Gray solid lines indicate estimated regression slope from LME model. B and C: Difference of PHE sensor and DBS over PHE sensor measurements (B) and DBS measurements (C). Upper and lower dashed lines are upper and lower tolerance limits. It can be concluded that extreme differences between PHE sensor and DBS measurements are a result of extreme PHE sensor measurements compared to corresponding DBS measurements. The x‐ and y‐axes are labeled from 0 to 2000 μmol/PHE and from −1000 to 2000 μmol/L.

Overall, the PHE sensor measured slightly higher than FIA‐MS/MS, with an average difference of 84.8 μmol/L (linear effects model, *p =* 0.0017). Median PHE levels were comparable between methods, with 556.9 μmol/L (range 48–1931 μmol/L) for the PHE sensor and 511.5 μmol/L (range 30–1586 μmol/L) for DBS. When divided into age‐dependent target ranges, the mean differences were 99.9 μmol/L for the range < 360 μmol/L (*N* = 116; 60%), 144.1 μmol/L for 360–600 μmol/L (*N* = 10; 5%), −19.3 μmol/L for 600–900 μmol/L (*N* = 15; 7.2%), and 41.9 μmol/L for 900–1200 μmol/L (*N* = 54; 27.8%). Large differences between PHE sensor and DBS FIA‐MS/MS measurements were a result of extreme PHE sensor measurements (Figure 3 and Figure S5).

**FIGURE 4:**
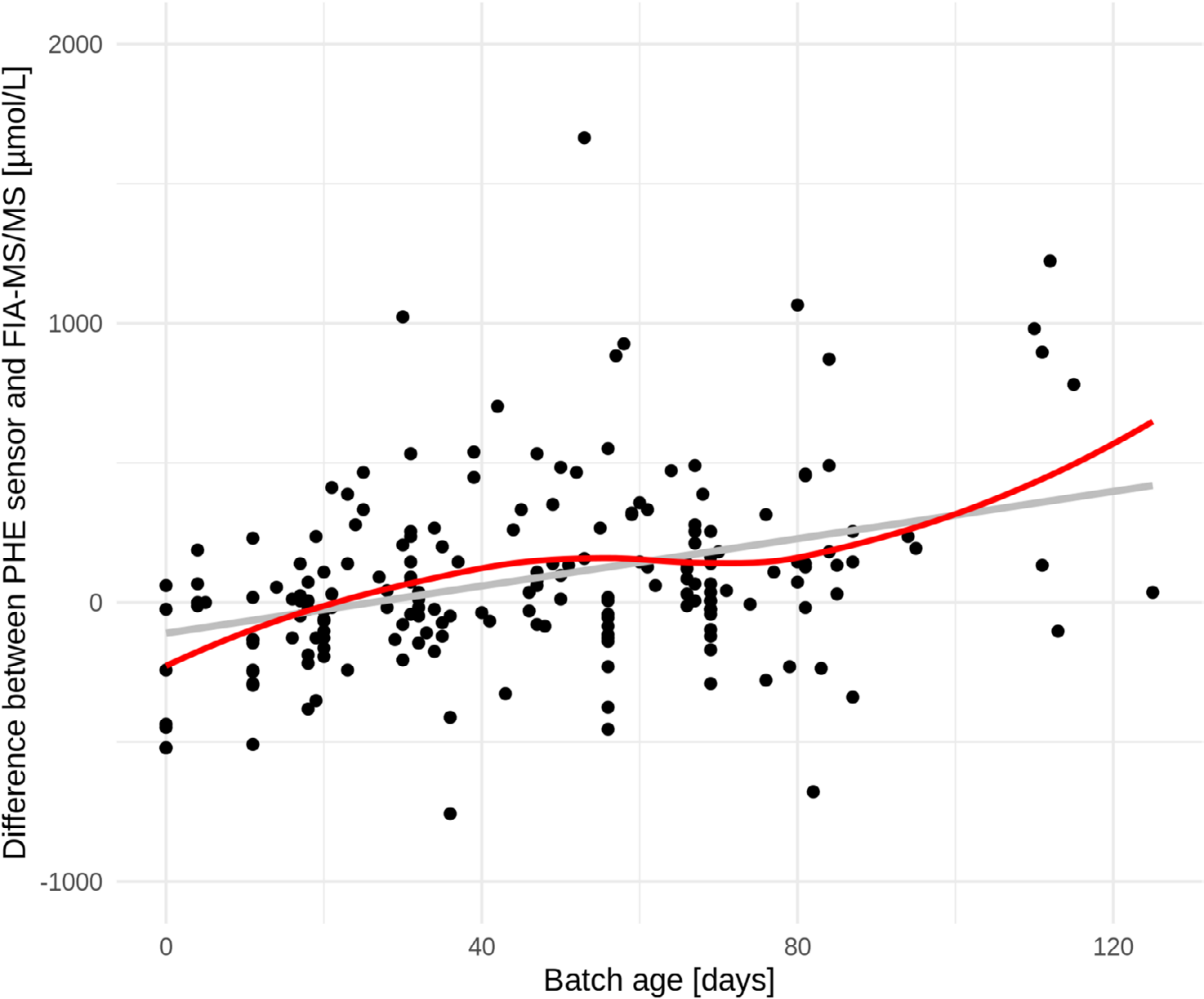
Difference of PHE sensor and DBS across age of batch. Regression line was estimated in LME model, solid red line shows a scatterplot smoothing function. The y‐axis is labeled from −1000 to 2000 μmol/L PHE.

Measurement differences increased with the PHE sensor batch age: the older the test batch, the greater the difference between the measurements (*β*_batch age_ = 0.0620; *p* = 0.001, LME) (Figure 4). The mean batch age of differences outside the upper tolerance limit was 77 days (median: 80 days).

At‐home measurements showed a larger variation (mean 146.6 μmol/L) compared to those from the first supervised test (mean −113.97 μmol/L) (*p* = 0.0016). Batches used in supervised tests were newer (mean 35 days) compared to at‐home tests (mean 50 days, *p* < 0.0001). After adjusting for batch age, supervised PHE sensor measurements showed significantly lower differences from DBS measurements than at‐home measurements (LME: *p* = 0.0001).

The differences between the devices (LME: *p* = 0.2018), age of participants (LME: *p* = 0.8805), and confidence in handling rated in the questionnaire (LME: *p* = 0.6087) are not significant. Also, the sex, batch number, and infection status have no significant impact on differences of the measurements (Table 2 and Table S4).

**TABLE 2:** Results from linear mixed effect models.

Subgroup analysis		Difference of values between PHE sensor and DBS (FIA‐MS/MS) in μmol/L			Linear mixed effects model analysis
		*N*	Mean	SD	
Differences across groups	Group 1	45	45.87	212.12	*p* = 0.1131
	Group 2	55	55.25	236.07	
	Group 3	44	144.05	424.56	
	Group 4	25	116.71	503.59	
	Group 5	25	83.78	278.65	
Differences across devices	Device 1	10	−130.76	386.4	*p* = 0.2018
	Device 3	12	−71.13	165.14	
	Device 4	10	−32.69	119.9	
	Device 5	10	56.3	457.58	
	Device 6	14	169.07	257.75	
	Device 7	16	−12.86	215.13	
	Device 8	7	41.51	159.65	
	Device 10	11	−30.82	313.98	
	Device 11	9	238.11	421.27	
	Device 13	19	66.27	200.25	
	Device 16	12	347.58	566.29	
	Device 17	8	260.31	348.49	
	Device 18	3	242.15	647.82	
	Device 19	15	50.04	168.95	
	Device 20	13	213.74	313.44	
	Device 21	5	−104.12	321.08	
	Device 22	7	26.81	200.8	
	Device 23	6	137.22	265.36	
	Device 24	7	266.36	469.45	
Differences across batch number	Number 11	9	−23.54	162.76	*p* = 0.6322
	Number 12	66	51.64	237.45	
	Number 14	25	76.28	209	
	Number 15	35	134.91	451.59	
	Number 16	32	116.91	447.46	
	Number 17	27	106.95	316.58	
Differences across sexes	Male	69	53.69	261.25	*p* = 0.5957
	Female	125	101.85	361.92	
Differences across infection status	Infection	38	136.84	303.37	*p* = 0.1791
	No infection	156	72.14	335.56	
Differences across supervised and at‐home tests	Supervised measurement (1st test)	46	−113.97	205.96	*p* = 0.0016
	At‐home measurements (2nd–5th tests)	148	146.6	337	

In 37 instances, FIA‐MS/MS values were within the age‐related target range, whereas the PHE sensor was not (Table 3). In these samples, the mean difference was 496.1 μmol/L, and the mean batch age was 62.1 days. There were significantly more false positive samples (43.02%), in which the PHE sensor value was outside the target range while the DBS value was within range, than false negatives samples (15.74%) in which the opposite was true (McNemar's *χ* ^2^ test with continuity correction: *p* = 0.009722). When analyzing only samples with a batch age below 77 days (165 measurements), the false positive rate reduced to 36.23%, with a mean difference of 428.36 μmol/L. The overall agreement between the two methods in classifying patients correctly into target versus out‐of‐target range was 72.2%. This aligns with the observed positive bias of the PHE sensor test, making it more likely to classify patients as above their target range.

**TABLE 3:** False‐positive measurements with the PHE sensor.

PHE sensor [μmol/L]	DBS FIA‐MS/MS [μmol/L]	Age‐dependent target range [μmol/L]	Batch age [days]
1222.8	811.2	< 1200	21
1392.3	1077.5	< 1200	76
1259.2	793	< 1200	25
1265.2	780.9	< 900	50
1362.1	871.7	< 900	84
520.6	339	< 360	70
538.8	84.8	< 360	81
629.6	96.9	< 360	31
496.4	357.2	< 360	17
399.5	260.3	< 360	66
429.8	284.5	< 360	80
417.7	211.9	< 360	30
472.2	236.1	< 360	31
435.9	290.6	< 360	31
478.2	266.4	< 360	67
405.6	260.3	< 360	87
1216.8	236.1	< 360	110
1804	139.2	< 360	53
550.9	230	< 360	59
672	284.5	< 360	68
411.6	357.2	< 360	14
696.2	339	< 360	60
1271.3	811.2	< 1200	81
1682.9	460.1	< 1200	112
1319.7	617.5	< 1200	42
1440.8	1180.5	< 1200	44
1931.1	1047.3	< 1200	57
1725.3	944.4	< 1200	115
1870.6	944.4	< 1200	58
1428.6	1162.3	< 1200	55
1216.8	1083.6	< 1200	111
1622.4	599.3	< 1200	30
1894.8	1023.1	< 1200	84
1713.2	817.2	< 1200	111
393.5	332.9	< 360	47
1289.4	224	< 360	80
514.6	181.6	< 360	61

*Note:* The table shows the 37 samples in which PHE sensor measurements were above the age‐dependent target range, while the corresponding FIA‐MS/MS measurements were within the target range.

### 3.3 Processing Time: PHE Sensor Versus DBS

The measurement time with the PHE sensor was approximately 15 min. This would also be the time it would take for the result to be available to the patient in the future. In comparison, DBS cards from at‐home measurements arrived at the laboratory after a median time of 2 days (range 0–11 days), and results with medical assessment were available after a median of 3 days (range 1–11 days) after the measurement date. During the study, the results were manually uploaded into the app; whereas, normally results would be sent by mail or communicated via phone.

### 3.4 Patient Questionnaire: Feasibility of the PHE Sensor and *Phenyx* App

Of the 47 participants, 46 (98%) completed the questionnaire (36% patients' mothers, 30% female adult patients, 12% fathers, 8% boys, 6% parents, 6% girls, 2% male adult patients).

Instructions during onboarding and the user manual were rated as “very good” (87%) and “good” (13%). Confidence during measurement was reported as “very confident” (37%), “confident” (46%), “mostly confident” (15%), or “okay” (2%). Confidence in handling increased with repeated use: 41% of participants felt confident after the first test, 48% after the second, and 9% after the third. However, 2% never felt fully confident, likely due to the monthly testing interval, suggesting a gradual learning process.

Individual steps of the test procedure were rated “very good” and “good” (median of 1–2). The waiting time until the blood sample was applied received a lower rating (median of 3, 67% “okay”, Figure S6). The design of the *phenyx* app was rated “very good” (41%), “good” (52%), and “okay” (7%). Overall handling of the PHE sensor was rated “very good” (56%), “good” (35%), and “satisfactory” (9%). Malfunction issues were reported by 36%, mostly related to the app, with frequent comments on Wi‐Fi interruptions.

**FIGURE 5:**
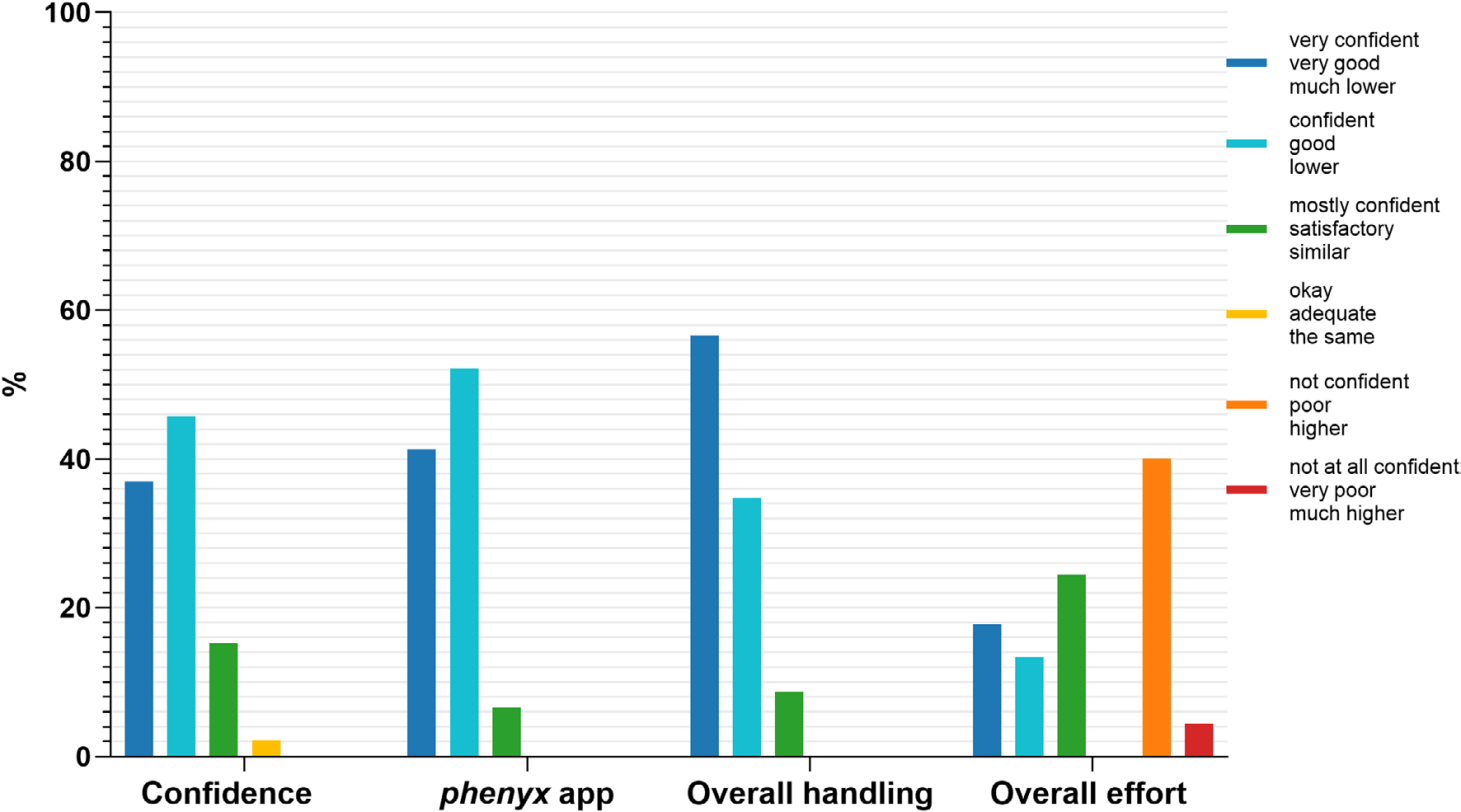
Ratings of confidence during the measurement, design of the *phenyx* app, overall handling of the PHE sensor, and comparison of the overall effort using the PHE sensor and *phenyx* app versus DBS. The bar chart shows the percentage distribution of ratings. *N* = 46 answered the questions on confidence, design of the *phenyx* app, and the overall handling. *N* = 45 answered the question on the overall effort.

While 40% rated the PHE sensor as requiring more effort and 4% much more effort than DBS testing (Figure 5), 100% of the participants preferred the PHE sensor over laboratory testing, and 98% would recommend it to other PKU patients.

A LME model showed no significant differences in ratings of confidence during the measurement, overall handling, design of the *phenyx* app, or overall effort among the different groups performing the measurement.

Suggested features for future app development included in‐app communication with healthcare providers and dietitians, a food diary and PHE intake tracker, visualization of additional amino acids, and reminders for measurements and appointments.

## 4 Discussion

With our clinical study we evaluated PoC measurement of PHE in a home setting in a large cohort of children and adults with PKU. We demonstrate that at‐home quantification of PHE using the PHE sensor in combination with the *phenyx* app is feasible, well accepted, and yields results comparable to the current laboratory standard of FIA‐MS/MS.

The PHE sensor showed a small positive bias compared to DBS FIA‐MS/MS (mean 84.8 μmol/L), which remained within acceptable limits for novel diagnostic technologies. The PHE sensor demonstrated high accuracy across clinically relevant ranges (< 360 to 1200 μmol/L), which is essential for PKU management where adherence to strict age‐specific target ranges is critical [2, 3, 4, 5, 6].

Unlike other biosensors that can only detect PHE levels up to approximately 435.9 μmol/L [13], the PHE sensor is able to quantify higher values relevant for older PKU patients. While enzyme‐based biosensors using PAH may be affected in PKU patients undergoing Pegvaliase therapy [1, 13], our NADPH‐based PHE sensor should remain unaffected.

A major clinical advantage of our home‐based PHE sensor testing is the rapid availability of results directly at‐home in 15 min compared to the median of 3 days for DBS results. This immediate feedback could enable timely dietary adjustments, which are particularly important for children and pregnant women, helping minimize unnecessary dietary restrictions and reduce psychosocial burden [1]. User acceptance was high, with most participants finding the PHE sensor and *phenyx* app easy to use and preferring them over laboratory testing, despite reporting a higher effort and a learning curve. The recently published at‐home PHE measurement method [19] also reported patient‐operated PHE testing with good agreement to the DBS standard (4.6% median variation), although the study population was more homogeneous, with 90% of samples ≤ 600 μmol/L, compared to 65% in our cohort. Additionally, the testing was conducted at home under professional supervision, with results available after 29 min. In contrast, our system was evaluated for independent home use, closer to the final use environment, and provided a shorter time‐to‐result. Practical aspects also differ, as our PHE sensor requires refrigeration, whereas the other system's test capsule is stored in the freezer and requires a thawing step before use.

Several limitations must be acknowledged. The overall sample size and observation period for validating a new device were modest. Self‐selection bias may also have favored more motivated users.

Another limitation relates to the reagent stability of the PHE sensor. Greater variability was observed with increasing batch age, with a functional shelf‐life calculated at 77 days under real‐life conditions. Test refrigeration and regular kit supply posed additional challenges, particularly during travel or in warmer climates. The stability of tube 2, containing lyophilized reagents, may have been affected by moisture absorption before sealing, manual sealing at ambient conditions, and inconsistencies in the lyophilization process. The lyophilizer also lacked integrated freezing and cycle logging, limiting process control. These factors likely contributed to increased batch variability and accelerated reagent degradation. Addressing these technical issues will require improved process control, optimized formulations, automated sealing in controlled environments, and comprehensive stability testing under different humidity and temperature conditions.

Greater variability was observed in at home compared to supervision testing. This could be attributed to user‐dependent factors such as incomplete capillary blood collection, insufficient mixing of reagents, or incomplete sample transfer. Smartphone positioning or handling errors might have also contributed to the variability, especially when combined with older batches or suboptimal storage. These findings suggest that extended patient training, a testing schedule with more frequent tests, as well as a simplified, more user‐friendly test design with limited user influence, could help minimize variability in at‐home settings.

In the current format, the PHE sensor relies on the integrated iPhone 14 Pro camera and an active internet connection. For broader accessibility, future versions should enable offline measurements and compatibility with a wide range of smartphones. Furthermore, the test should be more automated. Economic considerations will also be critical as reimbursement and costs have traditionally limited the adoption of new medical devices in rare and pediatric diseases [22]. However, integrating the system with patients' existing smartphones could provide a cost‐effective solution.

## 5 Conclusion

In conclusion, our findings support the use of the PHE sensor and *phenyx* app as a reliable and user‐friendly tool for home‐based PHE monitoring. Real‐world validation demonstrates that this integrated solution has the potential to enhance patient autonomy, improve metabolic control, and reduce both the clinical and psychological burden of PKU. Future studies should investigate long‐term use and integration into routine care pathways. Broader implementation could meaningfully transform PKU management by decentralizing care without compromising diagnostic quality. The further development and planned release of the app could facilitate communication between patients and healthcare providers in our increasingly digital world. Additionally, the *phenyx* app and the PHE sensor kit may be expandable to other inherited metabolic diseases with defined biomarkers, such as maple syrup urine disease, also requiring frequent laboratory measurements and communication between patients and healthcare providers for therapy adjustments.

## Author Contributions

Thomas Opladen and Anna T. Reischl‐Hajiabadi developed and supervised the clinical study. Kai Johnsson, Corentin Gondrand and Estelle Bonedeau conducted the laboratory analyses and the development of the PHE sensor. Nicolas Weiß, Gerd Schneider and Maurice Fahn developed the *phenyx* app. Frederic Myers and Philip Minel Städle contributed to the design of the *phenyx* app. Anna T. Reischl‐Hajiabadi, Thomas Opladen, Corentin Gondrand, Estelle Bonedeau, Nicolas Weiß and Kathrin Jeltsch contributed to the study design, coordination of the trial, and data collection. Sven F. Garbade performed statistical analyses. Dorothea Haas contributed to the data interpretation. Dorothea Haas and Jürgen G. Okun contributed to laboratory analyses of DBS with FIA‐MS/MS. Anna T. Reischl‐Hajiabadi, Corentin Gondrand, Estelle Bonedeau and Thomas Opladen wrote the first draft of the manuscript. All authors edited or substantively reviewed the publication and had full access to all the data in the study. The senior authors (Thomas Opladen and Kai Johnsson) had full access to the complete dataset of the study and had final responsibility for the decision to submit for publication. All authors approved the final manuscript as submitted and agree to be accountable for all aspects of the work.

## Funding

We gratefully acknowledge M. Lomtadze for his financial support of this study. Project support by the Fraunhofer‐Max Planck Cooperation Program and the European Union’s Horizon 2020 research and innovation program under the Marie Skłodowska‐Curie grant agreement No. 955623 (H2020‐ MSCA ITN‐CONSENSE). The authors confirm independence from the sponsors; the content of the article has not been influenced by the sponsors.

## Ethics Statement

This single‐center study (German Clinical Trials Register; trial registration ID: DRKS00031972) was approved by the local ethics committee (University Hospital Heidelberg, Germany; application number: S‐109/2023).

## Consent

All procedures followed were in accordance with the ethical standards of the responsible committee on human experimentation (institutional and national) and with the Helsinki Declaration of 1975, as revised in 2013. Informed consent was obtained from all patients and/or caregivers for being included in the study.

## Conflicts of Interest

Kai Johnsson is listed as inventor on patents on bioluminescent sensor proteins owned by MPG. All other authors declare no conflicts of interest.

## Supporting information


**Table S1:** Study schedule.
**Table S2:** Survey at the end of the study regarding the use of the PHE sensor and the *phenyx* app.
**Figure S1:** PHE measurement principle using the NADPH sensor.
**Figure S2:** Iphone device comparison.
**Figure S3:** PHE sensor calibration and inter‐batch variation.
**Figure S4:** Individual measurements of PHE sensor and DBS.
**Table S3:** Exclusion of PHE measurements and reasons.
**Figure S5:** Plot of tolerance.
**Table S4:** Results from linear mixed effect models. Difference of values between PHE sensor and DBS (FIA‐MS/MS) in μmol/L.
**Figure S6:** Ratings of the instructions and test steps.

## Data Availability

The data that support the findings of the study are available from the corresponding authors upon reasonable request.
